# Quaternary ammonium compounds (QAC) issues encountered in an environmental services (EVS) department

**DOI:** 10.1186/2047-2994-4-S1-P42

**Published:** 2015-06-16

**Authors:** L Sullivan, JM Boyce, A Brown, J Baker

**Affiliations:** 1 Yale-New Haven Hospital, New Haven, Connecticut, USA

## Introduction

QAC are the most common agents used in the United States for disinfection of healthcare surfaces. Recently, concern arose over the ability of cloths to bind QAC. Both microfiber cloths and cotton cloths are available in our facility.

## Objectives

This study evaluated the reduction of QAC concentrations with 3 types of cloths used for environmental disinfection, and identified variations in QAC concentrations delivered by dispensing stations.

## Methods

Three buckets were filled with a QAC solution measured at 800 parts per million (ppm). Thirty microfiber cloths were placed in one bucket, 30 cotton cloths in another, and a roll of disposable cloths in another. Three cloths were removed from each bucket every 5 minutes for the first 30 minutes, then every 30 minutes for a total time of 4 hours. At each time point, excess solution was wrung from the cloths and tested using QAC test paper and the average concentration of the solution expressed from 3 cloths was determined. In addition, a survey of 33 disinfectant dispensing stations was conducted to measure QAC concentrations delivered. Mixing stations are designed to dispense ½ ounce of concentrated QAC per gallon of water, yielding an in-use concentration of 800 ppm.

## Results

After cloths had been submerged in QAC for 5 minutes, the concentration of solution expressed from the 3 types of cloths was reduced by 21% with microfiber cloths and by 50% with cotton and disposable cloths. After 30 minutes the concentration of solution expressed from the cloths stablized and remained at that level for the following 3.5 hours: microfiber at 400 ppm, cotton at 200 ppm and disposable near zero. Of the 33 dispensing stations surveyed, concentrations measured <200 ppm from 7 stations, 200-400 ppm from 17 stations and 400-600 ppm from 6 stations. Two stations were found empty and 1 station inoperative. Investigation identified design flaws in dispensing stations.

## Conclusion

QAC binding varies with different types of cloths. Hospitals need to ensure the appropriate concentration of QAC solution is being dispensed and a cloth with low QAC binding properties is being used to ensure adequate surface disinfection is achieved.

## Disclosure of interest

L. Sullivan: None declared, J. Boyce Grant/Research support from: Virox., Conflict with: Scientific advisory board for 3M and Clorox., A. Brown: None declared, J. Baker: None declared.

